# A case of a four-year-old child adopted at eight months with unusual mood patterns and significant polypharmacy

**DOI:** 10.1186/s12888-017-1492-y

**Published:** 2017-09-11

**Authors:** Magdalena Romanowicz, Alastair J. McKean, Jennifer Vande Voort

**Affiliations:** Mayo Clinic, Department of Psychiatry and Psychology, 200 1st SW, Rochester, MN 55901 USA

**Keywords:** Case report, Adoption, Neglect, Polypharmacy, Disinhibited social engagement disorder

## Abstract

**Background:**

Long-term effects of neglect in early life are still widely unknown. Diversity of outcomes can be explained by differences in genetic risk, epigenetics, prenatal factors, exposure to stress and/or substances, and parent-child interactions. Very common sub-threshold presentations of children with history of early trauma are challenging not only to diagnose but also in treatment.

**Case presentation:**

A Caucasian 4-year-old, adopted at 8 months, male patient with early history of neglect presented to pediatrician with symptoms of behavioral dyscontrol, emotional dysregulation, anxiety, hyperactivity and inattention, obsessions with food, and attachment issues. He was subsequently seen by two different child psychiatrists. Pharmacotherapy treatment attempted included guanfacine, fluoxetine and amphetamine salts as well as quetiapine, aripiprazole and thioridazine without much improvement. Risperidone initiated by primary care seemed to help with his symptoms of dyscontrol initially but later the dose had to be escalated to 6 mg total for the same result. After an episode of significant aggression, the patient was admitted to inpatient child psychiatric unit for stabilization and taper of the medicine.

**Conclusions:**

The case illustrates difficulties in management of children with early history of neglect. A particular danger in this patient population is polypharmacy, which is often used to manage transdiagnostic symptoms that significantly impacts functioning with long term consequences.

## Background

There is a paucity of studies that address long-term effects of deprivation, trauma and neglect in early life, with what little data is available coming from institutionalized children [1]. Rutter [2], who studied formerly-institutionalized Romanian children adopted into UK families, found that this group exhibited prominent attachment disturbances, attention-deficit/hyperactivity disorder (ADHD), quasi-autistic features and cognitive delays. Interestingly, no other increases in psychopathology were noted [2].

Even more challenging to properly diagnose and treat are so called sub-threshold presentations of children with histories of early trauma [3]. Pincus, McQueen, & Elinson [4] described a group of children who presented with a combination of co-morbid symptoms of various diagnoses such as conduct disorder, ADHD, post-traumatic stress disorder (PTSD), depression and anxiety. As per Shankman et al. [5], these patients may escalate to fulfill the criteria for these disorders. The lack of proper diagnosis imposes significant challenges in terms of management [3].

## Case presentation

J is a 4-year-old adopted Caucasian male who at the age of 2 years and 4 months was brought by his adoptive mother to primary care with symptoms of behavioral dyscontrol, emotional dysregulation, anxiety, hyperactivity and inattention, obsessions with food, and attachment issues. J was given diagnoses of reactive attachment disorder (RAD) and ADHD. No medications were recommended at that time and a referral was made for behavioral therapy.

She subsequently took him to two different child psychiatrists who diagnosed disruptive mood dysregulation disorder (DMDD), PTSD, anxiety and a mood disorder. To help with mood and inattention symptoms, guanfacine, fluoxetine, methylphenidate and amphetamine salts were all prescribed without significant improvement. Later quetiapine, aripiprazole and thioridazine were tried consecutively without behavioral improvement (please see Table 1 for details).

**Table 1:** Review of patient’s current and historical medications

Indication	Medication	Dose range	Patient’s age when used
ADHD	Guanfacine	0.25–2 mg	2 yrs. 5mo-4 yrs. (no effect)
	Methylphenidate (Ritalin)	5–10 mg	3 yrs. (tried for less than a month due to lack of effect)
	Amphetamine salts (Adderall)	5 mg	4 yrs. (started at the hospital, tried for less than a month due to increase in aggression and agitation)
Insomnia	Melatonin^a^	2.5–10 mg at bedtime	2 yrs. 5mo-present (worked initially, currently no effect)
	Topiramate	50 mg at bedtime	3 yrs. (tried for less than a month, stopped by neurology due to concerns for interactions with Depakote)
Behavioral dyscontrol	Risperidone^a^	0.1– 6 mg	2 yrs. 8mo-4 yrs. (tapered off during psychiatric hospitalization due to lack of effect)
	Aripiprazole	1–10 mg	2 yrs. 8mo (stopped as no effect).
	Quetiapine	150–300 mg	3 yrs. (tried for about 3 months)
	Thioridazine	10 mg	3 yrs. (tried for less than a month)
Anxiety	Fluoxetine	5–10 mg	3 yrs. (tried for less than a month)
Seizures	Depakote^a^	375–500 mg	3 yrs.-present (continued during the hospitalization)

^a^Medicines on admission

No significant drug/substance interactions were noted (Table 1). There were no concerns regarding adherence and serum drug concentrations were not ordered. On review of patient’s history of medication trials guanfacine and methylphenidate seemed to have no effect on J’s hyperactive and impulsive behavior as well as his lack of focus. Amphetamine salts that were initiated during hospitalization were stopped by the patient’s mother due to significant increase in aggressive behaviors and irritability. Aripiprazole was tried for a brief period of time and seemed to have no effect. Quetiapine was initially helpful at 150 mg (50 mg three times a day), unfortunately its effects wore off quickly and increase in dose to 300 mg (100 mg three times a day) did not seem to make a difference. Fluoxetine that was tried for anxiety did not seem to improve the behaviors and was stopped after less than a month on mother’s request.

J’s condition continued to deteriorate and his primary care provider started risperidone. While initially helpful, escalating doses were required until he was on 6 mg daily. In spite of this treatment, J attempted to stab a girl at preschool with scissors necessitating emergent evaluation, whereupon he was admitted to inpatient care for safety and observation. Risperidone was discontinued and J was referred to outpatient psychiatry for continuing medical monitoring and therapy.

Little is known about J’s early history. There is suspicion that his mother was neglectful with feeding and frequently left him crying, unattended or with strangers. He was taken away from his mother’s care at 7 months due to neglect and placed with his aunt. After 1 month, his aunt declined to collect him from daycare, deciding she was unable to manage him. The owner of the daycare called Child Services and offered to care for J, eventually becoming his present adoptive parent.

J was a very needy baby who would wake screaming and was hard to console. More recently he wakes in the mornings anxious and agitated. He is often indiscriminate and inappropriate interpersonally, unable to play with other children. When in significant distress he regresses, and behaves as a cat, meowing and scratching the floor. Though J bonded with his adoptive mother well and was able to express affection towards her, his affection is frequently indiscriminate and he rarely shows any signs of separation anxiety.

At the age of 2 years and 8 months there was a suspicion for speech delay and J was evaluated by a speech pathologist who concluded that J was exhibiting speech and language skills that were solidly in the average range for age, with developmental speech errors that should be monitored over time. They did not think that issues with communication contributed significantly to his behavioral difficulties. Assessment of intellectual functioning was performed at the age of 2 years and 5 months by a special education teacher. Based on Bailey Infant and Toddler Development Scale, fine and gross motor, cognitive and social communication were all within normal range.

J’s adoptive mother and in-home therapist expressed significant concerns in regards to his appetite. She reports that J’s biological father would come and visit him infrequently, but always with food and sweets. J often eats to the point of throwing up and there have been occasions where he has eaten his own vomit and dog feces. Mother noticed there is an association between his mood and eating behaviors. J’s episodes of insatiable and indiscriminate hunger frequently co-occur with increased energy, diminished need for sleep, and increased speech. This typically lasts a few days to a week and is followed by a period of reduced appetite, low energy, hypersomnia, tearfulness, sadness, rocking behavior and slurred speech. Those episodes last for one to 3 days. Additionally, there are times when his symptomatology seems to be more manageable with fewer outbursts and less difficulty regarding food behaviors.

J’s family history is poorly understood, with his biological mother having a personality disorder and ADHD, and a biological father with substance abuse. Both maternally and paternally there is concern for bipolar disorder.

## Diagnosis

J has a clear history of disrupted attachment. He is somewhat indiscriminate in his relationship to strangers and struggles with impulsivity, aggression, sleep and feeding issues. In addition to early life neglect and possible trauma, J has a strong family history of psychiatric illness. His mood, anxiety and sleep issues might suggest underlying PTSD. His prominent hyperactivity could be due to trauma or related to ADHD. With his history of neglect, indiscrimination towards strangers, mood liability, attention difficulties, and heightened emotional state, the possibility of Disinhibited Social Engagement Disorder (DSED) is likely. J’s prominent mood lability, irritability and family history of bipolar disorder, are concerning for what future mood diagnosis this portends.

As evidenced above, J presents as a diagnostic conundrum suffering from a combination of transdiagnostic symptoms that broadly impact his functioning. Unfortunately, although various diagnoses such as ADHD, PTSD, Depression, DMDD or DSED may be entertained, the patient does not fall neatly into any of the categories.

## Discussion

This is a case report that describes a diagnostic conundrum in a young boy with prominent early life deprivation who presented with multidimensional symptoms managed with polypharmacy.

A sub-threshold presentation in this patient partially explains difficulties with diagnosis. There is no doubt that negative effects of early childhood deprivation had significant impact on developmental outcomes in this patient, but the mechanisms that could explain the associations are still widely unknown. Significant family history of mental illness also predisposes him to early challenges. The clinical picture is further complicated by the potential dynamic factors that could explain some of the patient’s behaviors. Careful examination of J’s early life history would suggest such a pattern of being able to engage with his biological caregivers, being given food, being tended to; followed by periods of neglect where he would withdraw, regress and engage in rocking as a self-soothing behavior. His adoptive mother observed that visitations with his biological father were accompanied by being given a lot of food. It is also possible that when he was under the care of his biological mother, he was either attended to with access to food or neglected, left hungry and screaming for hours.

The current healthcare model, being centered on obtaining accurate diagnosis, poses difficulties for treatment in these patients. Given the complicated transdiagnostic symptomatology, clear guidelines surrounding treatment are unavailable. To date, there have been no psychopharmacological intervention trials for attachment issues. In patients with disordered attachment, pharmacologic treatment is typically focused on co-morbid disorders, even with sub-threshold presentations, with the goal of symptom reduction [6]. A study by dosReis [7] found that psychotropic usage in community foster care patients ranged from 14% to 30%, going to 67% in therapeutic foster care and as high as 77% in group homes. Another study by Breland-Noble [8] showed that many children receive more than one psychotropic medication, with 22% using two medications from the same class.

It is important to note that our patient received four different neuroleptic medications (quetiapine, aripiprazole, risperidone and thioridazine) for disruptive behaviors and impulsivity at a very young age. Olfson et al. [9] noted that between 1999 and 2007 there has been a significant increase in the use of neuroleptics for very young children who present with difficult behaviors. A preliminary study by Ercan et al. [10] showed promising results with the use of risperidone in preschool children with behavioral dyscontrol. Review by Memarzia et al. [11] suggested that risperidone decreased behavioral problems and improved cognitive-motor functions in preschoolers. The study also raised concerns in regards to side effects from neuroleptic medications in such a vulnerable patient population. Younger children seemed to be much more susceptible to side effects in comparison to older children and adults with weight gain being the most common. Weight gain associated with risperidone was most pronounced in pre-adolescents (Safer) [12]. Quetiapine and aripiprazole were also associated with higher rates of weight gain (Correll et al.) [13].

Pharmacokinetics of medications is difficult to assess in very young children with ongoing development of the liver and the kidneys. It has been observed that psychotropic medications in children have shorter half-lives (Kearns et al.) [14], which would require use of higher doses for body weight in comparison to adults for same plasma level. Unfortunately, that in turn significantly increases the likelihood and severity of potential side effects.

There is also a question on effects of early exposure to antipsychotics on neurodevelopment. In particular in the first 3 years of life there are many changes in developing brains, such as increase in synaptic density, pruning and increase in neuronal myelination to list just a few [11]. Unfortunately at this point in time there is a significant paucity of data that would allow drawing any conclusions.

## Conclusions

Our case report presents a preschool patient with history of adoption, early life abuse and neglect who exhibited significant behavioral challenges and was treated with various psychotropic medications with limited results. It is important to emphasize that subthreshold presentation and poor diagnostic clarity leads to dangerous and excessive medication regimens that, as evidenced above is fairly common in this patient population.

Neglect and/or abuse experienced early in life is a risk factor for mental health problems even after adoption. Differences in genetic risk, epigenetics, prenatal factors (e.g., malnutrition or poor nutrition), exposure to stress and/or substances, and parent-child interactions may explain the diversity of outcomes among these individuals, both in terms of mood and behavioral patterns [15–17]. Considering that these children often present with significant functional impairment and a wide variety of symptoms, further studies are needed regarding diagnosis and treatment.

## Abbreviations

ADHD: Attention-Deficit/Hyperactivity Disorder
DMDD: Disruptive Mood Dysregulation Disorder
DSED: Disinhibited Social Engagement Disorder
PTSD: Post-Traumatic Stress Disorder
RAD: Reactive Attachment disorder
